# Alterations in airway microbiota in patients with PaO_2_/FiO_2_ ratio ≤ 300 after burn and inhalation injury

**DOI:** 10.1371/journal.pone.0173848

**Published:** 2017-03-30

**Authors:** Dana M. Walsh, Shaun D. McCullough, Scott Yourstone, Samuel W. Jones, Bruce A. Cairns, Corbin D. Jones, Ilona Jaspers, David Diaz-Sanchez

**Affiliations:** 1 Curriculum in Toxicology, University of North Carolina, Chapel Hill, North Carolina, United States of America; 2 National Health and Environmental Effects Research Laboratory, U.S. Environmental Protection Agency, Chapel Hill, North Carolina, United States of America; 3 Department of Biology, University of North Carolina, Chapel Hill, North Carolina, United States of America; 4 Program in Bioinformatics and Computational Biology, University of North Carolina, Chapel Hill, North Carolina, United States of America; 5 Department of Surgery, University of North Carolina, Chapel Hill, North Carolina, United States of America; 6 North Carolina Jaycee Burn Center, University of North Carolina, Chapel Hill, North Carolina, United States of America; 7 Curriculum in Genetics and Molecular Biology, University of North Carolina, Chapel Hill, North Carolina, United States of America; 8 Lineberger Comprehensive Cancer Center, University of North Carolina, Chapel Hill, North Carolina, United States of America; 9 Carolina Center for Genome Sciences, University of North Carolina, Chapel Hill, North Carolina, United States of America; 10 Department of Pediatrics, University of North Carolina, Chapel Hill, North Carolina, United States of America; 11 Center for Environmental Medicine, Asthma, and Lung Biology, University of North Carolina, Chapel Hill, North Carolina, United States of America; Wageningen Universiteit, NETHERLANDS

## Abstract

**Background:**

Injury to the airways after smoke inhalation is a major mortality risk factor in victims of burn injuries, resulting in a 15–45% increase in patient deaths. Damage to the airways by smoke may induce acute respiratory distress syndrome (ARDS), which is partly characterized by hypoxemia in the airways. While ARDS has been associated with bacterial infection, the impact of hypoxemia on airway microbiota is unknown. Our objective was to identify differences in microbiota within the airways of burn patients who develop hypoxemia early after inhalation injury and those that do not using next-generation sequencing of bacterial 16S rRNA genes.

**Results:**

DNA was extracted from therapeutic bronchial washings of 48 patients performed within 72 hours of hospitalization for burn and inhalation injury at the North Carolina Jaycee Burn Center. DNA was prepared for sequencing using a novel molecule tagging method and sequenced on the Illumina MiSeq platform. Bacterial species were identified using the MTToolbox pipeline. Patients with hypoxemia, as indicated by a PaO_2_/FiO_2_ ratio ≤ 300, had a 30% increase in abundance of *Streptococcaceae* and *Enterobacteriaceae* and 84% increase in *Staphylococcaceae* as compared to patients with a PaO_2_/FiO_2_ ratio > 300. Wilcoxon rank-sum test identified significant enrichment in abundance of OTUs identified as *Prevotella melaninogenica (p* = 0.042), *Corynebacterium* (*p* = 0.037) and *Mogibacterium* (*p* = 0.048). Linear discriminant effect size analysis (LefSe) confirmed significant enrichment of *Prevotella melaninognica* among patients with a PaO_2_/FiO_2_ ratio ≤ 300 (*p*<0.05). These results could not be explained by differences in antibiotic treatment.

**Conclusions:**

The airway microbiota following burn and inhalation injury is altered in patients with a PaO_2_/FiO_2_ ratio ≤ 300 early after injury. Enrichment of specific taxa in patients with a PaO_2_/FiO_2_ ratio ≤ 300 may indicate airway environment and patient changes that favor these microbes. Longitudinal studies are necessary to identify stably colonizing taxa that play roles in hypoxemia and ARDS pathogenesis.

## Introduction

Smoke-induced inhalation injury occurs in up to 43% of burn victims, increasing death rates by up to 20% as compared to patients with burn injury alone [1]. Inhalation injury predisposes these patients to respiratory failure, acute respiratory distress syndrome (ARDS), and pneumonia. Pneumonia, in combination with burn and inhalation injury, further increases patient mortality to 60% and is a contributing risk factor to development of ARDS [2,3]. ARDS is a life-threatening condition resulting from either direct or indirect injury to the lung, and is diagnosed clinically by the presence of bilateral opacities on chest imaging and airway hypoxemia [3,4]. Hypoxemia is determined by the ratio of the partial pressure of arterial oxygen (PaO_2_) to the fraction of inspired oxygen (FiO_2_). To meet the Berlin definition of ARDS, this ratio must be less than or equal to 300 mm Hg, with a minimum positive end expiratory pressure (PEEP) of 5 cm H_2_O [4]. Although bacterial infection is frequently the first step towards pneumonia and sepsis, and can induce direct injury to the lung and contribute to the pathogenesis of ARDS, its relationship with the disease is complex and not well understood [5].

Early antibiotic therapy is critical to improved patient outcomes once infection and pneumonia occur, but identification of the organisms can be challenging [6]. Current methodologies rely on culture or polymerase chain reaction (PCR) techniques to identify the causative agent [7]; however, these methods require specific knowledge of the organism’s growth and metabolic requirements and a period of 1–2 days for identification and susceptibility testing, which are prone to false positive results [7]. These limitations often result in broad-spectrum antibiotic treatment that may have little impact on the target organism, promote the development of antibiotic resistance, and ultimately increase mortality [6,7].

To address the limitation of organism identification, we utilized next-generation sequencing of bacterial 16S rRNA genes to characterize the bacterial communities (collectively known as microbiota) in the airways of burn patients following smoke inhalation with or without a PaO_2_/FiO_2_ ratio ≤ 300, regardless of the presence of ARDS. Study of the microbiota has revealed the key roles they play in the development and function of the host immune system, and how dysbiosis, or perturbation of the communities, contributes to disease [8–10]. Although host-microbiota interactions are complex and poorly understood, recent studies underscore the importance of low-abundance species in dysbiosis and disease progression, particularly in the airways [10,11]. We hypothesized that inhalation injury and a low P/F ratio (≤ 300) would create conditions within the airways that favor distinct communities of bacteria. We show that facultative anaerobic taxa are enriched among all burn patients, and that specific, low-abundance bacterial taxa are associated with low P/F ratios within the first 24 to 72 hours after injury.

## Methods

### Patients and sample collection

Therapeutic bronchial washings from patients hospitalized for burn and inhalation injury at the North Carolina Jaycee Burn Center were collected as previously described [12]. Briefly, patients with suspected inhalation injury underwent clinically indicated bronchoscopy within 24 hours of admission. All patients were intubated, bronchial washes performed, and inhalation injury severity scored on the basis of examination. Only those patients for whom inhalation injury was confirmed by bronchoscopy were included in the present study. Only samples taken within three days of injury were utilized. Clinical cultures were grown to detect bacteria within these bronchoscopy samples. Organisms detected per patient and antibiotic treatment are listed in S3 Table in the additional data. Differential cell counts were not done for the bronchial washings. According to the Berlin definition of ARDS, hypoxemia was defined as the ratio of the partial pressure of arterial oxygen (PaO_2_) to the fraction of inspired oxygen (FiO_2_) ≤300 [4,12]. Ratios >300 were defined as normal oxygenation levels [3]. The P/F ratio for each patient in this study was measured the same day the bronchial washing was done. Other clinical information, such as patient demographics and total body surface area burned, were collected upon admission. The study protocol was approved by the Institutional Review Board at the University of North Carolina School of Medicine in Chapel Hill (IRB# 10–0959 and #12–2475). All patients or their legally authorized representative gave written informed consent for collection of their bronchial washings for inclusion in a repository as previously described [12]. Analysis of the microbiota in bronchial washings was not an original part of the study and was added after completion of sample collection (IRB #12–2475).

### DNA extraction and sequencing

Bronchial washes were transported on ice and processed within 24–48 hours. DNA was extracted from the cellular portion of the wash and quantified (online data supplement). Positive *Staphylococcus aureus* and negative reagent and human DNA controls were extracted simultaneously and prepared in parallel with the patient samples for sequencing. Sequencing of all DNA was performed in duplicate by a molecule tagging method recently described by Lundberg et al., [13]. This approach allows us to confidently identify operational taxonomic units (OTU) that diverge at the 3% threshold. Briefly, a short round of polymerase chain reaction (PCR) was performed to attach molecule tags to each DNA molecule, followed by a round of full PCR to label each individual sample with a barcode and attach the adapters necessary for sequencing. The primers targeted the V4 region of the bacterial 16S rRNA gene with forward sequence GTGCCAGCMGCCGCGGTAA (515F) and reverse sequence TAATCTWTGGGVHCATCAGG (806R) [13]. Sequencing was performed on the Illumina MiSeq platform at the High Throughput Sequencing Facility at the University of North Carolina at Chapel Hill.

### Sequencing data and statistical analysis

We used the MTToolbox [14] pipeline to minimize sequencing errors and match reads to the GreenGenes 16S rRNA database [15]. Sequences that did not match a 16S GreenGenes sequence were removed from the OTU table and those remaining were corrected for variation among 16S rRNA operon number. An R-squared read number correlation was performed on technical replicates in order to determine an appropriate threshold of low read count OTUs to remove from the data [16]. This method improves reproducibility of the results while minimizing loss of data. Total raw counts per OTU of duplicate patient samples were averaged and count thresholds were set for the OTU tables using an R-squared correlation analysis as detailed previously [16]. The samples varied according to the date of sequencing and thresholds were set separately for each group (S1 and S2 Figs). Samples from patients with and without hypoxemia were distributed among the sequencing plates as shown in S1 Table. OTU tables with appropriate thresholds were imported into the program Explicet [17] for normalization and subsequent diversity analyses and the Wilcoxon rank-sum and two-proportions tests. Rarefaction was performed on sample counts within Explicet before bootstrapping to calculate Chao1 diversity indices. The Wilcoxon test is a non-parametric, continuity-corrected test appropriate for analysis of differential OTU abundances [17]. The two-proportions test performs a continuity-adjusted chi-square test to determine differences in detection among OTUs [17]. One-way analysis of variance (ANOVA) was used to identify differences among the abundance of aerobic and anaerobic bacterial taxa present in patients with and without a P/F ratio ≤ 300 (performed in R as anova = lm(Taxa_per_seq_count~Group, data = ALI)) [18]. The phyloseq package within R was used to create a principle components analysis (PCA) plot to compare beta diversity across patients [19]. The linear discriminant analysis (LDA) effect size (LEfSe) method [20] was used to determine the significance of differences in taxa abundance by biologically relevant classes, which included patient P/F ratio and antibiotic treatment. LEfSe first performs a factorial Kruskal-Wallis test to determine differential distribution of OTUs among the biological classes. If subclasses are present, a pairwise Wilcoxon test is done on those with *p* values greater than 0.05. OTUs with significant differences are then used to build a linear discriminant analysis model, which uses the relative differences of OTUs among classes to rank those that are most discriminative. To determine the influence of antibiotic treatment and sequencing batch effect on these results, we performed a non-parametric differential abundance analysis adjusted for antibiotic treatment and batch effects [21].

## Results

### Patients

Of the 48 patients included in this study, 50% had P/F ratios ≤ 300 (Table 1). Of the 24 patients with P/F ratios > 300, 7 subsequently had a P/F ratio < 300. All patients who did not survive had initial P/F ratios < 300. The rate of positive bacterial cultures in both patients with (21%) and without (25%) a P/F ratio ≤ 300 was similar to the overall rate (23%). However, the rate of antibiotic treatment within the first 72 hours of injury in patients with a P/F ratio ≤ 300 was lower (29%) than either patients with a higher P/F ratio (46%) or the entire group (40%). Antibiotic treatment was not associated with P/F ratio ≤ 300 (chi-square test, *p* = 0.4).

**Table 1:** Patient clinical characteristics.

Clinical Variable	Total	PaO_2_/FiO_2_ ≤ 300	PaO_2_/FiO_2_ > 300	T Test *p* Value
Patients	48	24	24	NA
Males	36 (75%)	18 (75%)	18 (75%)	NA
Females	12 (25%)	6 (25%)	6 (25%)	NA
BMI	27 (14–51)	30 (17–51)	25 (14–42)	0.1107
Age	41 (1–75)	42 (8–76)	41 (1–75)	0.8143
%TBSA	19 (0–85)	27 (0–85)	10 (0–40)	0.002
Antibiotic Treated	18 (40%)	7 (29%)	11 (46%)	0.3711^¶^
Baux Score	60 (1–115)	71 (31–115)	50 (1–96)	0.0109
Endotracheal Tube	29 (60%)	17 (71%)	12 (50%)	0.3099^¶^
Days on Ventilator	35 (0–105)	45 (0–105)	25 (0–79)	0.0136
Positive Cultures	11 (23%)	5 (21%)	6 (25%)	0.894^¶^
Survived	41 (87%)	17 (71%)*	24 (100%)	0.02497^¶^

Patient clinical characteristics were grouped by total population and subdivided by P/F ratio. The data are represented as mean (range) or number (percent). PaO_2_/FiO_2_ > 300 and PaO_2_/FiO_2_ ≤ 300 group percentages are calculated per group total.

*Cause of death was either or both cardiac and pulmonary failure.

^¶^Indicates the *p* value from Pearson’s chi-squared test.

%TBSA = percent total body surface area burn.

### The airway microbiota among all patients

**Fig 1:**
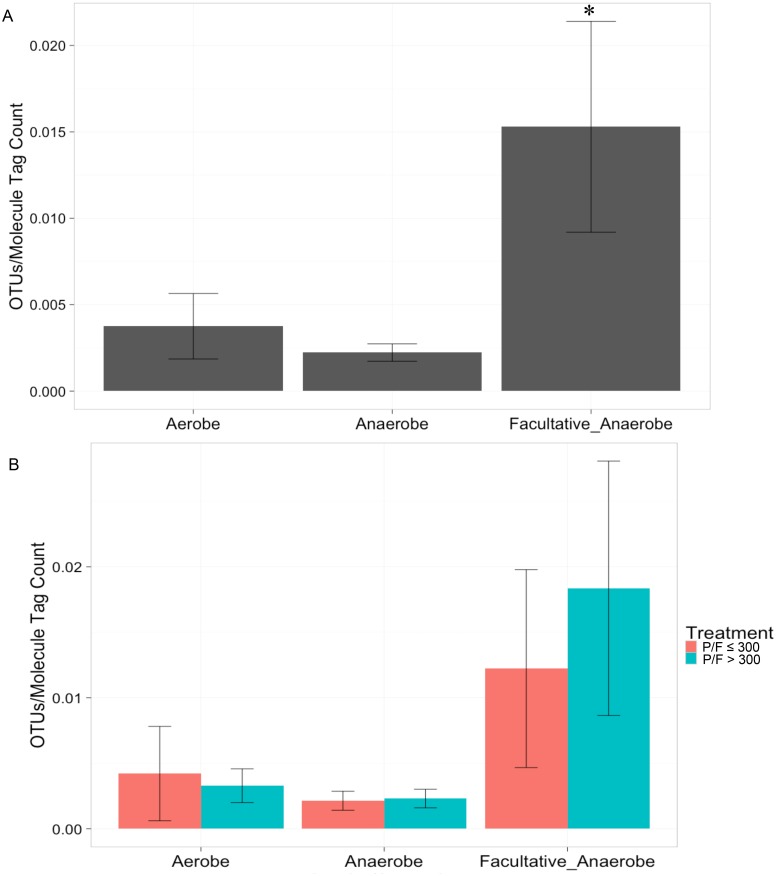
Increased detection of unique facultative anaerobic taxa. (A) Unique facultative anaerobic OTUs were detected significantly more frequently than obligate aerobes or anaerobes among all patients. (B) No significant difference was found between number of unique facultative anaerobic OTUs when the data was split by patient PaO_2_/FiO_2_ ratio. OTUs were identified as facultative anaerobes, obligate anaerobes, or obligate aerobes among all patients. OTUs were quantified and normalized to the molecule tag count and averaged by bacterial aerobic or anaerobic capability. One-way ANOVA detected a significant difference among the mean taxa of facultative anaerobes (*p* = 0.029). (n = 48)

**Fig 2:**
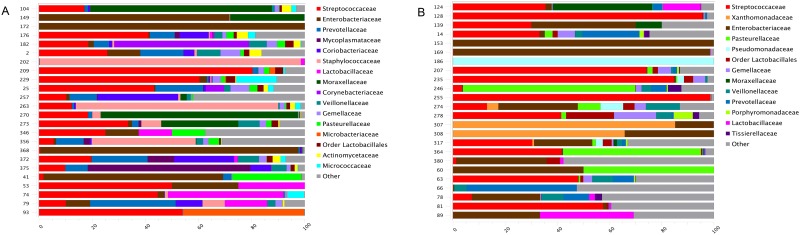
Patient airway microbiota composition. OTUs identified as the families Streptococcaceae and Enterobacteriaceae dominate the airway microbiota within 72 hours following burn and inhalation injury. (A) The microbiota composition among patients with hypoxemia. (B) The microbiota composition among patients without hypoxemia. Each horizontal bar represents an individual patient microbiota normalized to 100%. Different colored sections within each bar indicate abundance of specific families. The category ‘Other’ includes bacterial taxa present at less than 1% of the total community. (n = 24)

Among all patient samples, OTUs identified as facultative anaerobic taxa were detected at a significantly higher rate than OTUs identified as either obligate anaerobic or obligate aerobic taxa (Fig 1A; ANOVA *p* = 0.029). When we split the data by PaO_2_/FiO_2_ ratio, patients with PaO_2_/FiO_2_ >300 contained more unique facultative anaerobic OTUs than those with PaO_2_/FiO_2_ ≤ 300, but this difference was not significant (Fig 1B). The most abundant OTUs among all patient samples at the family level were *Streptococcaceae* and *Enterobacteriaceae*, which accounted for 26% and 18% of total family-level OTUs, respectively. The remaining 56% of OTUs consisted of 45 additional families, each present at 7% of the total family-level OTUs or less. Fig 2 shows the composition of the microbiota per patient for those with (Fig 2A) and without (Fig 2B) hypoxemia. Positive and negative control samples were sequenced with the patient samples and S2 Table quantifies the total reads and molecule tags produced and S3 Fig shows the composition of these reads.

### Enrichment of low-abundance OTUs among patients with PaO_2_/FiO_2_ ≤ 300

**Fig 3:**
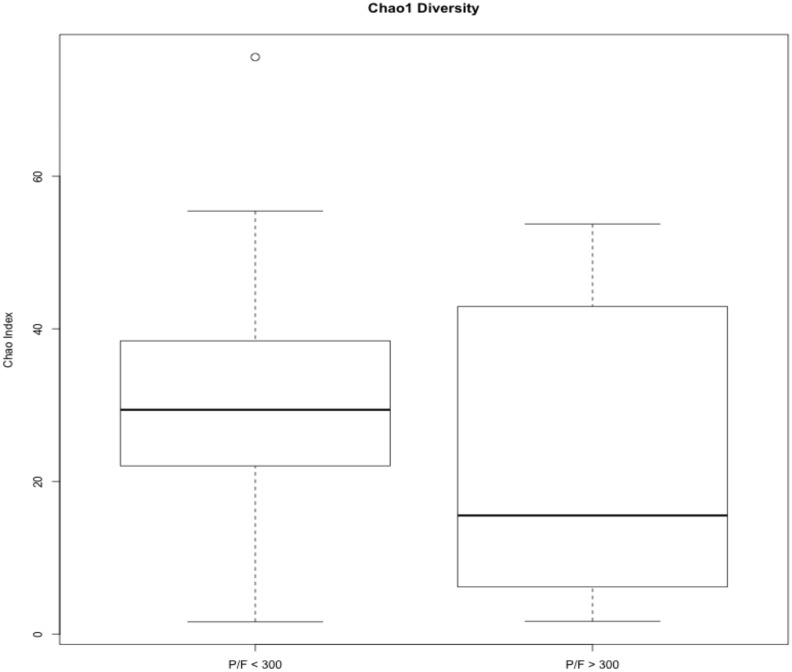
Chao1 diversity among patients with and without hypoxemia. Samples were rarefied prior to calculation of the Chao1 diversity index and averaged based on PaO_2_/FiO_2_ ratio. Student’s T test did not show significant differences between the patient groups. (n = 48)

The *Streptococcaceae* and *Enterobacteriaceae* family-level OTU abundances were not significantly different between patients with and without PaO_2_/FiO_2_ ≤ 300 (Wilcoxon test, *p* >0.05). At the lowest level of OTU identification, *Enterobacteriaceae* family-level OTUs, *Streptococcus* genus-level OTUs, and *Staphylococcus* genus-level OTUs were detected in 80% of patients both with and without PaO_2_/FiO_2_ ≤ 300 (Table 2; the average abundance refers to the percent of each OTU within each group). However, when compared to patients with PaO_2_/FiO_2_ > 300, patients with PaO_2_/FiO_2_ ≤ 300 had a 27% increase in OTUs identified as *Streptococcus spp*., a 32% increase in Enterobacteriaceae, and an 83% increase in *Staphylococcus spp*, calculated as the percent change. An additional six OTUs were detected in 80% of patients with PaO_2_/FiO_2_ ≤ 300 at 3.1% or less of the total OTUs in this group (Table 2). All OTUs detected in 80% of patients were either facultative or obligate anaerobes. Figs 2 and 3 display OTU abundances at the family level that account for greater than 1% of the total OTUs among individual patients without and with PaO_2_/FiO_2_ ≤ 300, respectively.

**Table 2:** Average abundance of taxa detected among 80% of patients.

Bacteria	Aerobe/Anaerobe	Average Abundance Among Patients with PaO_2_/FiO_2_ > 300*	Average Abundance Among Patients with PaO_2_/FiO_2_ ≤ 300^§^
OTU10: *Enterobacteriaceae*	Facultative anaerobe	20.0	17.0
OTU75: *Streptococcus spp*.	Facultative anaerobe	27.8	22.1
OTU58: *Staphylococcus spp*	Facultative anaerobe	2.7	9.2
OTU35: *Atopobium spp*.	Facultative anaerobe	NA	3.1
OTU62: *Gemellaceae*	Facultative anaerobe	NA	1.8
OTU108: *Veillonella dispar*	Obligate anaerobe	NA	1.2
OTU65: *Lactobacillales*	Facultative anaerobe	NA	0.7
OTU45: *Prevotella spp*.	Obligate anaerobe	NA	2.4
OTU47: *Prevotella melaninogenica*	Obligate anaerobe	NA	2.5

Taxa detected in 80% of patients with and without PaO_2_/FiO_2_ ≤ 300. Taxa names represent the lowest level of identification of the corresponding OTU.

*Percent of total OTUs among 24 patients with PaO_2_/FiO_2_ > 300.

^§^Percent of total OTUs among 24 patients with PaO_2_/FiO_2_ ≤ 300.

NA indicates that these OTUs were not present among 80% of patients with PaO_2_/FiO_2_ > 300.

### Alpha diversity among patients with and without PaO_2_/FiO_2_ ≤ 300

The Chao1 diversity index, which is a non-parametric species richness estimator [22], did not show significant differences in number of different OTUs between patients with and without PaO_2_/FiO_2_ ≤ 300 (Fig 3). Though the median Chao1 index in patients with PaO_2_/FiO_2_ > 300 is less than that of patients with PaO_2_/FiO_2_ ≤ 300, it shows a much broader range in patients with PaO_2_/FiO_2_ > 300.

### Beta diversity among patients with and without PaO_2_/FiO_2_ ≤ 300

**Fig 4:**
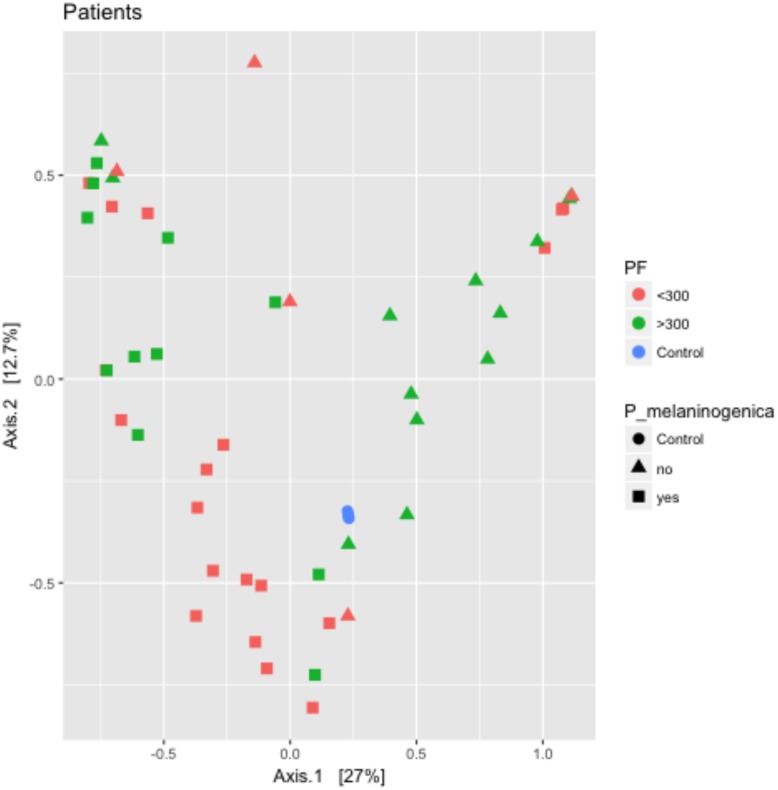
Clustering by principle components analysis suggests microbiota similarity in the presence of *P*. *melaninogenica*. The R package phyloseq was used to generate a PCA plot of patient microbiota abundance. Patient samples containing *P*. *melaninogenica* cluster to the left side of the graph and are split based on PaO_2_/FiO_2_ ratio. Manhattan distance was used to perform the ordination prior to plotting the PCA graph. Data points are colored by patient PaO_2_/FiO_2_ ratio (Red: P/F < 300; Green: P/F > 300) and data point shape indicates presence (square) or absence (triangle) of *P*. *melaninogenica*. Control samples are included for comparison (blue circles). (n = 48).

The PCA plot in Fig 4 displays similarity of microbiota among burn patients with inhalation injury. Data points represent individual patients, which are colored by PaO_2_/FiO_2_ ratio. The shape of each data point represents whether *P*. *melaninogenica* was detected in the community. Patients with PaO_2_/FiO_2_ ratios > 300 with *P*. *melaninogenica* present cluster to the left above the horizontal zero axis on the graph, while those with PaO_2_/FiO_2_ ratios < 300 with *P*. *melaninogenica* cluster to the left below it. Patients with PaO_2_/FiO_2_ ratios > 300 without *P*. *melaninogenica* are more spread out along the right side of the graph while those with PaO_2_/FiO_2_ ratios < 300 and without *P*. *melaninogenica* are scattered throughout. This clustering pattern suggests that the presence of *P*. *melaninogenica* is associated with microbiota similarity regardless of PaO_2_/FiO_2_ ratio, while in the absence of this microbe a PaO_2_/FiO_2_ ratio higher than 300 may influence similarity. Low PaO_2_/FiO_2_ ratio without *P*. *melaninogenica* does not appear to be associated with microbiota similarity.

### Significant enrichment of specific OTUs among patients with PaO_2_/FiO_2_ ≤ 300

**Fig 5:**
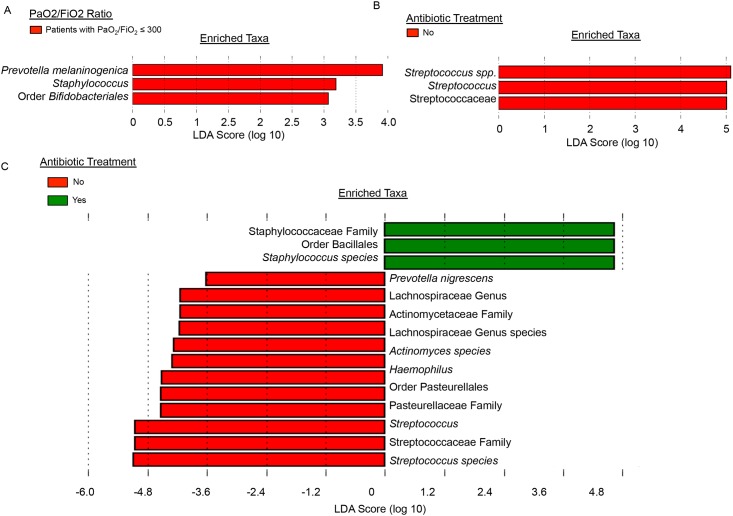
Significant enrichment of specific taxa. Specific bacterial taxa are enriched among patients with PaO_2_/FiO_2_ ≤ 300. (A) LEfSe analysis detected significant enrichment of OTUs identified as *Prevotella melaninogenica*, *Staphylococcus spp*., and the order Bifidobacteriales among patients with hypoxemia. No significant enrichment was detected among patients without hypoxemia. (B) Among only those patients with hypoxemia, only those not treated with antibiotics contained significantly enriched taxa, all of which were in the Streptococcaceae family. No significant enrichment of taxa was detected among patients not treated with antibiotics in this comparison. (C) Antibiotic treatment alters the microbiota among all patients, with a specific increase in *Staphylococcus* among patients treated with antibiotics, but does not impact association of the *Prevotella melaninogenica* OTU with hypoxemia. LEfSe uses a Kruskal-Wallis rank-sum test, Wilcoxon rank-sum test, and linear discriminant analysis to determine the biological relevance of significant enrichment of taxa and ranks them by effect size. LDA score indicates the magnitude of the effect size.

**Fig 6:**
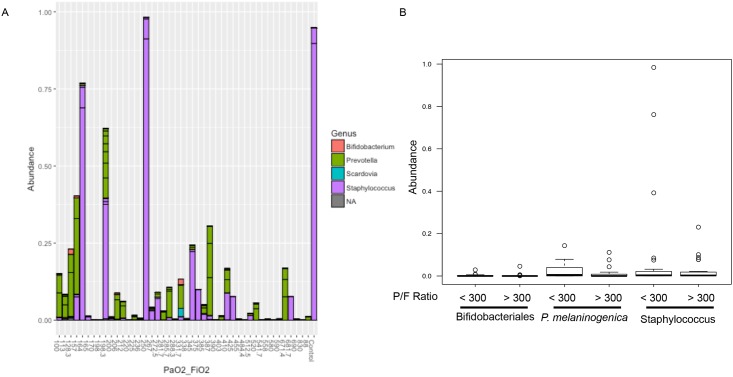
Percent abundance of OTUs with significant enrichment detected by LEfSe. Of the three taxa LEfSe identified as significantly enriched in patients with hypoxemia, *Staphylococcus* had the highest percent increase in abundance. However, *Prevotella melaninogenica* was more consistently present among patients with hypoxemia, resulting in its higher ranking over *Staphylococcus spp*. (A) Bacterial abundances for taxa identified as significantly enriched by LEfSe are displayed per patient. The X axis is labeled with each patient’s PaO_2_/FiO_2_ ratio and the Y axis displays relative abundance of the taxa. (B) The range of abundances of the three significantly enriched taxa are split by patient hypoxemia status along the X axis. (n = 48).

Four OTUs were identified as significantly different in abundance and detection between patients with and without PaO_2_/FiO_2_ ≤ 300. OTUs identified as *Prevotella melaninogenica*, *Mogibacterium spp*., and *Corynebacterium spp*. were significantly increased in abundance among patients with PaO_2_/FiO_2_ ≤ 300 (Table 3). Patients with PaO_2_/FiO_2_ ≤ 300 had 72% more of the OTU represented by *Prevotella melaninogenica* than patients with ratios > 300, 79% more *Corynebacterium* genus-level OTU, and 86% more of the *Mogibacterium* genus-level OTU. *Prevotella melaninogenica* OTUs were also detected significantly more frequently among patients with PaO_2_/FiO_2_ ≤ 300, while *Corynebacterium* OTUs were significantly more frequent in patients with ratios > 300 (Table 4). OTUs identified as *Fusobacterium spp*. were also detected significantly more frequently among patients with PaO_2_/FiO_2_ ≤ 300. LEfSe [20] was used to confirm these results. LEfSe identified the *Prevotella melaninogenica* OTU as most discriminative among patients with PaO_2_/FiO_2_ ≤ 300 as compared to those with ratios > 300, followed by *Staphylococcus* genus-level and then *Bifidobacteriales* order-level OTUs (Fig 5A). Though *Staphylococcus* OTUs are more abundant among the patients (Fig 6A), their average abundance within patients with hypoxemia is less than that of *P*. *melaninogenica* (Fig 6B). *Prevotella* is consistently present among the patients (Fig 6A) and its average abundance in patients with hypoxemia is greater than that for the other two significantly enriched taxa (Fig 6B). Additional analysis with LEfSe indicated significant enrichment of *Staphylococcus spp*. OTUs in the presence of antibiotics, while enrichment of *Prevotella melaninogenica* OTUs was not affected (Fig 5B and 5C). Among all patients in the study, LEfSe identified significant enrichment of taxa only in patients with hypoxemia (Fig 5A). Among only patients with hypoxemia, LEfSe found significant enrichment of taxa only in patients treated with antibiotics (Fig 5B). LEfSe identified significant enrichment of taxa in patients that did and did not receive antibiotics when all patients were included in the analysis (Fig 5C). Among patients without hypoxemia, several bacterial taxa were enriched with and without antibiotic treatment (S5 Fig). Non-parametric differential abundance analysis adjusted for antibiotic treatment and sequencing batch effect confirmed that enrichment of *P*. *melaninogenica* was not affected by antibiotic treatment or batch effect.

**Table 3:** Percent abundance OTU level differences.

Taxa	Percent Abundance Among Patients with PaO_2_/FiO_2_ ≤ 300	Percent Abundance Among Patients with PaO_2_/FiO_2_ > 300	P-Value
OTU47: *Prevotella melaninogenica*	1.56 (0.04)	0.44 (7.7x10^-3^)	0.042
OTU18: *Corynebacterium spp*.	1.53 (0)	0.32 (1.8x10^-3^)	0.037
OTU82: *Mogibacterium spp*.	0.07 (1.0x10^-3^)	0.01 (1.3x10^-4^)	0.048

OTU level significant differences in abundance as determined by Wilcoxon rank-sum test. Taxa names represent the lowest level of identification of the corresponding OTU. Values are percent abundance (interquartile range).

**Table 4:** OTU level detection differences.

Taxa	Detection Rate Among Patients with PaO_2_/FiO_2_ ≤ 300 (# of patients)	Detection Rate Among Patients with PaO_2_/FiO_2_ > 300 (# of patients)	P-Value
OTU47: *Prevotella melaninogenica*	19	11	0.037
OTU18: *Corynebacterium spp*.	6	14	0.040
OTU115: *Fusobacterium spp*.	17	9	0.043

OTU level significant differences in detection as determined by the two-proportions test. Taxa names represent the lowest level of identification of the corresponding OTU.

## Discussion

Our work details differences in the airway microbiota in patients with PaO_2_/ FiO_2_ ratios ≤ 300 and > 300, following burn and inhalation injury. A cut-off of 300 was chosen based on the Berlin definition of airway hypoxemia in ARDS [4]. We identify several low-abundance OTUs with significant enrichment in patients with PaO_2_/FiO_2_ ≤ 300, of which the OTU identified as *Prevotella melaninogenica* was the most significant. In addition, we show that while antibiotic treatment alters the airway microbiota, it does not explain the enrichment of a specific OTU among patients with PaO_2_/FiO_2_ ≤ 300.

Patients with a PaO_2_/ FiO_2_ ratio that was less than or equal to 300 within 72 hours of burn and inhalation injury had consistently worse indicators of poor prognosis. Table 1 shows the average values for patients with and without PaO_2_/FiO_2_ ≤ 300 for several clinical variables that are predictive of injury severity. In patients with inhalation injury, several studies have demonstrated that age, percent TBSA and PaO_2_/ FiO_2_ ratio predict mortality [2]. The PaO_2_/ FiO_2_ ratio itself has been shown to be more predictive of patient outcomes on the day after patients meet the Berlin definition of ARDS rather than the day of [23]. In our study, patients with PaO_2_/FiO_2_ ≤ 300 within 72 hours of injury had, on average, a higher Baux score (age + %TBSA), spent longer on the ventilator, were intubated more frequently, and had lower survival rates. Only percent TBSA and the PaO_2_/ FiO_2_ ratio were significantly different among the patient groups (Student’s t test, *p* = 0.002 and 5.293e-11, respectively). Patients were 41 years old on average, but ranged from 1 to 75 years. Though not statistically more prevalent in this cohort, patients at the ends of this spectrum are more susceptible to infection, pneumonia, and poor outcomes [24]. Over a lifetime, a patient will range from increased susceptibility to infection after burn and inhalation injury, to decreased susceptibility in mid-life, to increased in old age. While fewer patients with PaO_2_/FiO_2_ ≤ 300 received antibiotic treatment than those with ratios > 300, rates of positive clinical bacterial cultures were similar between the two groups. This discrepancy may be partly due to the challenges in predicting bacterial infection and development of pneumonia in this patient population. Pneumonia is the primary complication of inhalation injury [25] and early, adequate antibiotic treatment has been shown to improve outcomes in these patients [6]. Criteria to predict pneumonia early after injury have been developed and include age > 60 years, TBSA > 20%, and initial PaO_2_/ FiO_2_ ratio of ≤ 300 [26]. The patients with PaO_2_/FiO_2_ ≤ 300 in our study meet the TBSA and initial PaO_2_/ FiO_2_ ratio criteria, but not the age criteria, which may explain why they did not receive as many antibiotics. A major limitation of this scoring system is its failure to take into account bacteria within the airways, emphasizing the need for one that does, perhaps through a combination of clinical cultures and next-generation sequencing of bacterial 16S rRNA genes.

Though we have focused on the PaO_2_/FiO_2_ ratio in alterations of the airway microbiota, TBSA may also contribute to the differences we detected. Increasing TBSA is a known predictor of patient mortality [25], which is compounded in the presence of inhalation injury. Burns greater than 20% TBSA induce systemic changes similar to those seen in trauma and surgical patients [27]. The injury induces a systemic inflammatory response, but compromises global immune function, increasing susceptibility to bacterial, viral, and fungal infections. Patients with PaO_2_/FiO_2_ ≤ 300 in our study had, on average, 27% TBSA, indicating immune dysfunction that could predispose them to airway bacterial colonization and infection. Though we cannot determine whether the burn injury itself induces PaO_2_/FiO_2_ ≤ 300 through systemic changes or if this is a direct result of inhalation injury, it is clear that TBSA may be contributing indirectly to alterations in the airway microbiota in our patient population. A mouse model of burn and inhalation injury is necessary to determine the extent to which TBSA influences changes in the airway microbiota.

Among all patients in the study, there were significantly more unique OTUs identified as facultative anaerobes than either strict anaerobes or aerobes (Fig 1A). Anaerobic taxa are normally associated with mucosal surfaces, but may lead to infection following disruption by trauma and surgery [28]. All patients within this study, regardless of PaO_2_/FiO_2_ ratio, presumably experienced disruption of their mucosa through the double trauma of burn and inhalation injury. Recent work has demonstrated that the mouth serves as the primary source community for the airway microbiota [29]. Inhalation injury may have increased microbial immigration through disruption of the mouth and upper airways’ mucosal surface, dislodging taxa that subsequently traveled down the airways to the bronchi. Alteration of airway conditions by inhalation injury may have selected for enrichment of facultative anaerobic taxa among all patients, which was significantly different from strict aerobic and anaerobic taxa (ANOVA, *p* = 0.029). When we subdivided the data by PaO_2_/FiO_2_ ratio, we did not see significant differences in strict aerobes, anaerobes or facultative anaerobes between the two patient groups (Fig 1B, *p* > 0.05). These results suggest that PaO_2_/FiO_2_ ≤ 300 early after burn and inhalation injury does not select for overall taxa in the airways based on their aerobic or anaerobic capabilities, but that burn and inhalation injury do. Development of PaO_2_/FiO_2_ ≤ 300 within 72 hours of burn and inhalation injury may not be enough time to observe significant change in the abundances of overall taxa between the two groups.

We detected OTUs identified as *Enterobacteriaceae*, *Streptococcus spp*., and *Staphylococcus spp*. in 80% of patients with and without PaO_2_/FiO_2_ ≤ 300 (Table 2). All three of these OTUs are facultative anaerobes and their dominance across patients implies similarity in the mechanism of injury to the airways selecting for these taxa and their related functions. Inhalation injury may induce fluctuations in oxygen availability in the airways, perhaps creating both aerobic and anaerobic microenvironments that favor taxa that can withstand these changes. Our finding of overall significant enrichment of facultative anaerobic taxa supports this idea. Patients with PaO_2_/FiO_2_ ≤ 300 demonstrated a 32%, 27%, and 83% increase in *Enterobacteriaceae*, *Streptococcus spp*., and *Staphylococcus spp*. OTUs, respectively, when compared to those with PaO_2_/FiO_2_ > 300 (Table 2). Additionally, 80% of patients with PaO_2_/FiO_2_ ≤ 300 contained six more OTUs that represented 3.1% and less of the total community among these patients (Table 2). This suggests that, although facultative anaerobes are enriched over strict anaerobes and aerobes among all patients, there are differences in enrichment of specific, low-abundance OTUs depending on PaO_2_/FiO_2_ ratio.

*Enterobacteriaceae*, *Streptococcus spp*., and *Staphylococcus spp*. have all been consistently detected in previous airway microbiome studies in both healthy and diseased airways [30]. Members of the *Enterobacteriaceae* family have been implicated in inflammation-driven colorectal cancer in the gut microbiome [31,32], are enriched in patients with COPD and asthma, but can also be detected in healthy airways [33–35]. Similarly, *Streptococcus* is consistently found in healthy airways but is enriched in COPD [35], idiopathic pulmonary fibrosis (IPF) [36], and pneumonia [37]. *Staphylococcus*, while a normal commensal in the nasal microbiome [38,39], is largely associated with disease in the lung, such as IPF [36], and cystic fibrosis, in which it is correlated with increased inflammation [40,41]. Given the inconsistency with which these three taxa are associated with health or disease, it is difficult to interpret the importance of their detection across patients with and without PaO_2_/FiO_2_ ≤ 300. They may indicate an underlying core airway microbiota among all burn and inhalation injury patients but it is not clear whether their presence is beneficial or detrimental. Given that sampling was done within 72 hours of injury, some of the taxa we have detected could be acquired nosocomial pathogens rather than commensal organisms, implying a detrimental impact. A longitudinal study of patients with burn and inhalation injury could clarify the role of these taxa.

Due to its association with health outcomes, overall diversity has long been a focus in many microbiome studies; however, we observed no difference in alpha diversity between patients with PaO_2_/FiO_2_ ≤ 300 and those with PaO_2_/FiO_2_ > 300 (Fig 3). Despite this, the PCA plot in Fig 4 suggests similarity in the microbiota between patients depending first on whether *P*. *melaninogenica* is present and then on patient PaO_2_/FiO_2_ ratio. Patients with *P*. *melaninogenica* clustered to the left side of the graph and were largely split by PaO_2_/FiO_2_ ratio. Patients without *P*. *melaninogenica* and PaO_2_/FiO_2_ ratio > 300 tended to cluster together, but those without this microbe and PaO_2_/FiO_2_ ratio < 300 were scattered throughout the graph. This agrees with recent studies demonstrating that diversity (especially in the airways) is a complex, multifactorial trait that encompasses more than an indication of positive or negative outcomes [34,41]. Many of these studies have emphasized the critical roles of specific taxa during disease and their interactions with other taxa [10,11]. They suggest that rare and less abundant taxa, which are overlooked by traditional culture methods, may play significant roles in the development of disease. Dysbiosis of the microbiota is followed by enrichment of a specific bacterial taxa that is either rarely found or present at very low abundance [10,37]. Changes in the balance of bacterial taxa alters how the microbes interact with each other along with their associated functions, allowing species that may have been suppressed by the presence of other bacteria to overgrow [10]. What was considered a harmless commensal in a healthy individual may become a harmful pathogen under dysbiosis-inducing conditions [42]. Accordingly, in our study, we observed significant differences not in the species dominating the overall community, but in less abundant taxa. While these taxa do not differ in microbial diversity between patient groups, they may differ by functional diversity, which ultimately plays a greater role in patient outcomes [30]. Most significantly, we identified enrichment of the OTU *Prevotella melaninogenica* among patients with PaO_2_/FiO_2_ ≤ 300 within 72 hours of burn and inhalation injury (Fig 5A).

*Prevotella melaninogenica* is a gram-negative obligate anaerobe that is part of the normal microbiota but is also a significant source of infection [43]. The specifics of *Prevotella melaninogenica*’s function in the microbiome remain unclear. In the gut, it has been identified as a normal commensal family, but within dental plaque it is a potential pathogen [44]. In the upper airways, the presence of *Prevotella melaninogenica* is associated with health while lactobacilli, *Rothia spp*., and *Streptococcus pneumoniae* dominate bacterial profiles in patients with pneumonia [37]. *Prevotella melaninogenica*’s positive role in the airways is supported by its ability to decrease production of T cell-activating IL-12p70 by dendritic cells exposed to *Haemophilus influenzae* [45]. This highlights the ability of bacteria within microbial communities to regulate each other’s functions as well as that of the host immune system. Several studies indicate that *Prevotella melaninogenica* could also play a non-beneficial or harmful role in the airways under certain conditions. *Prevotella melaninogenica* was a dominant bacterial species isolated from the airways of intubated patients [46] as well as cystic fibrosis (CF) patients, where characterized species varied phenotypically over time [47]. *Prevotella* species in CF airways have been shown to be virulent and contribute to the pathophysiology of the disease [48]. *Prevotella melaninogenica* specifically produces short-chain fatty acids that generate IL-8 production by host epithelial cells, presumably drawing neutrophils to the airways that contribute to the inflammatory status of the patient [49]. Though present at low abundance, we identified a consistent and significant enrichment of the *Prevotella melaninogenica* OTU among patients with PaO_2_/FiO_2_ ≤ 300 within 72 hours of burn and inhalation injury. While facultative anaerobic taxa were enriched among all patients in the study, *Prevotella melaninogenica* was enriched specifically in patients whose airways have the lowest PaO_2_/FiO_2_ ratio, which may select for growth of this obligate anaerobe. Without pre-injury samples from the patients, it is not possible to determine whether enrichment of *Prevotella melaninogenica* precedes PaO_2_/FiO_2_ ≤ 300 or if a low PaO_2_/FiO_2_ ratio precedes this enrichment. If confirmed in a longitudinal study, the consistent presence of this OTU throughout the hospital stay of patients with PaO_2_/FiO_2_ ≤ 300 would suggest that it is in some way altering the airway environment to favor *Prevotella melaninogenica*. This could be achieved through elimination of other OTUs that *Prevotella melaninogenica* interacts with that cannot thrive in hypoxic conditions or outgrowth of those that can. Early changes in both oxygen availability and other OTUs may impact *Prevotella melaninogenica*’s ability to act as a pathogen depending on whether species it interacts with are increased or eliminated or airway conditions alter its growth and pathogenicity. Given that *Prevotella melaninogenica* is an obligate anaerobe, hypoxic conditions may favor its growth, but it is impossible to predict its pathogenicity without further study. While determining a causal link between PaO_2_/FiO_2_ ≤ 300 and *Prevotella melaninogenica* is beyond the scope of the current study, future studies will examine its pathogenicity from patients with and without PaO_2_/FiO_2_ ≤ 300 as well as its role in either preceding or following hypoxemia.

Infection is a serious concern in these immunocompromised patients, for whom mortality rates increase to 20% with inhalation injury alone and triple to 60% when present with pneumonia [50]. Prophylactic antibiotic treatment is a common strategy to prevent infection, but results in as many as 25% of patients without infections receiving antibiotics [51], which may alter the microbiota in deleterious ways and encourage outgrowth of resistant bacteria [52]. Antibiotic treatment has been shown to perturb the gut microbiome and immune cell response by eliminating commensal species and allowing drug-resistant bacteria to take over [53,54]. In the airways, antibiotic treatment in asthma shows a similar response, in which elimination of certain species provides a niche for establishment of other infectious species [55]. In COPD, three months of varying types of antibiotic treatment in patients did not reduce overall bacterial load but instead increased antibiotic resistance across all groups [56]. While a powerful tool for controlling bacterial growth, antibiotic treatment is a double-edged sword that can create communities of bacteria resistant to treatment. Our poor understanding of bacterial interactions within the microbiota and their roles in patient outcomes combined with antibiotics’ lack of specificity results in overkilling of beneficial organisms that could aid in improving patient outcomes. In our study, 18 total patients were treated with antibiotics; 9 of these had negative culture results and for 2, cultures were not done (S3 Table). If negative culture results indicate absence of infection in these patients, antibiotic treatment is unnecessarily altering the airway microbiota, possibly contributing to development of resistance and poor outcomes. Among all patients treated with antibiotics, analysis with LEfSe indicated significant enrichment of bacteria in the *Staphylococcaceae* family and the order *Bacillales (*Fig 5C). These bacteria may be resistant to the drugs or not targeted by them, leading to overgrowth of these particular species. Methicillin-resistant *Staphylococcus aureus* is a known problematic infection in hospitals, including the Jaycee Burn Center, but its role within burn patient microbiota is unknown and requires further study. Despite alteration of other taxa by antibiotic treatment, enrichment with the *Prevotella melaninogenica* OTU among patients with PaO_2_/FiO_2_ ≤ 300 was not affected, implying that its association with hypoxemia is independent of antibiotic treatment, at least within 72 hours of injury (Fig 5B and 5C). Further study is necessary to determine the role of this OTU in early development of hypoxemia and whether targeted antibiotic treatment may be beneficial.

There are several limitations to the study. Although unique in its examination of a heterogeneous group of burn patients, our work is also limited by this variability. The heterogeneity of clinical diagnoses in this group makes interpretation of results challenging. We were unable to extract significantly more DNA from healthy human bronchial washings than we did from our negative reagent controls, and therefore were unable to include healthy control samples for comparison (S4 Fig). However, the number of patients studied is comparable to or larger than previous studies of microbiota in airway disease [10,35]. Longitudinal samples were taken throughout the course of each patient’s hospital stay, but the variation in drug treatments (including antibiotics) and therapies precluded achievement of statistical significance among the microbiota detected in these samples. Despite these limitations, this study is pioneering in its examination of the injured airway microbiota among burn patients and its association with patient outcomes.

In conclusion, we have demonstrated differences in the airway microbiota of patients with and without PaO_2_/FiO_2_ ≤ 300 within 72 hours of burn and inhalation injury. We detected overall enrichment of facultative anaerobes among all patients with differences in specific OTUs among patients with and without PaO_2_/FiO_2_ ≤ 300. Significant differences between these patients reside among the less abundant OTUs, specifically the *Prevotella melaninogenica* OTU, an obligate anaerobe whose role in the microbiome is unclear. Hypoxic conditions indicative of ARDS development may favor *Prevotella melaninogenica* enrichment and alter its pathogenicity. Alternatively, hypoxemia may develop due to increased abundance of this OTU following inhalation injury. A mouse model of inhalation injury is needed to determine whether development of hypoxemia drives enrichment of *Prevotella melaninogenica* or enrichment of this OTU induces hypoxemia. Given the cross-sectional nature of this study, more work is necessary to determine the long-term impact of *Prevotella melaninogenica* and its role in the airway microbiome of burn patients with inhalation injury who develop PaO_2_/FiO_2_ ≤ 300 within 72 hours of injury. Importantly, antibiotic treatment did not alter this association, supporting a link between this OTU and PaO_2_/FiO_2_ ≤ 300 early after burn and inhalation injury.

## Supporting information

S1 Fig Technical replicate comparison to determine sample groups for count threshold setting. Variability was minimized by grouping samples by the plate on which they were sequenced. The Y axis displays log10 transformed raw read counts per OTU for the first replicate and the X axis displays these values for the second replicate. (A) Log10 transformed raw counts per OTU from Plate 2, samples sequenced January 2015. (B) Log10 transformed raw counts per OTU from Plate 1, samples sequenced January 2015. (C) Log10 transformed raw counts per OTU from Plate 3, samples sequenced December 2014.
(TIF)

S2 Fig R-squared correlation of the progressive drop-out analysis used to set count thresholds by plate on which the samples were sequenced. The threshold for log10 transformed raw counts per OTU per sequencing batch was set where the regression began to plateau (red line), indicating acceptable levels of read count correlation between the replicate samples. (A) R^2^ values for raw counts for samples on Plate 2, samples sequenced January 2015. (B) R^2^ values for raw counts for samples on Plate 1, samples sequenced January 2015. (C) R^2^ values for raw counts for samples on Plate 3, samples sequenced December 2014.
(TIF)

S3 Fig Percent abundance of control OTUs. Each bar represents OTUs detected among human (16HBE), *Staphylococcus aureus* (SAUR), and reagent (CNTRL) DNA controls normalized to 100%. *n* = 6
(TIF)

S4 Fig No significant detection of bacterial DNA in healthy airway controls. Bronchoscopy was performed on healthy volunteers and DNA was extracted from airway washings in the same manner as the burn patient samples. Extracted DNA was quantified using the universal primer set developed by Maeda *et*. *al*. DNA extracted from *Staphylococcus aureus* and *Klebsiella pneumoniae* were used as positive controls and DNA from the human cell line 16HBE was used as a negative control. A water-only reagent control was included as well. DNA extracted from six healthy volunteers did not contain significantly more DNA than the negative control. (n = 6).
(TIF)

S5 Fig Significant enrichment of taxa among patients without hypoxemia in the presence or absence of antibiotic treatment. Specific taxa are enriched among non-hypoxemic patients who did and did not receive antibiotics. Analysis with LEfSe detects significant enrichment of bacteria in the Enterobacteriales order with antibiotic treatment, while several other taxa were enriched without treatment.
(TIF)

S1 Table Distribution of patient samples by PaO_2_ /FiO_2_ ratio among sequencing plates. (DOCX)

S2 Table Averaged percent of sequences for positive and negative controls. (DOCX)

S3 Table Patient clinical cultures. (DOCX)

S1 File Supporting Information File. (DOCX)

## Abbreviations

ANOVA: analysis of variance
ARDS: acute respiratory distress syndrome
CF: cystic fibrosis
DNA: deoxyribonucleic acid
FiO_2_: fraction of inspired oxygen
IPF: idiopathic pulmonary fibrosis
LEfSe: linear discriminant analysis effect size
OTU: operational taxonomic unit
PaO_2_: partial pressure of arterial oxygen
PCA: principle components analysis
PCR: polymerase chain reaction
rDNA: ribosomal deoxyribonucleic acid
