# Supplementary figures and images for: Alterations in airway microbiota in patients with PaO2/FiO2 ratio ≤ 300 after burn and inhalation injury
Source: PLoS One. 2017 Mar 30;12(3):e0173848. doi: 10.1371/journal.pone.0173848 (PMC5373524; doi:10.1371/journal.pone.0173848)

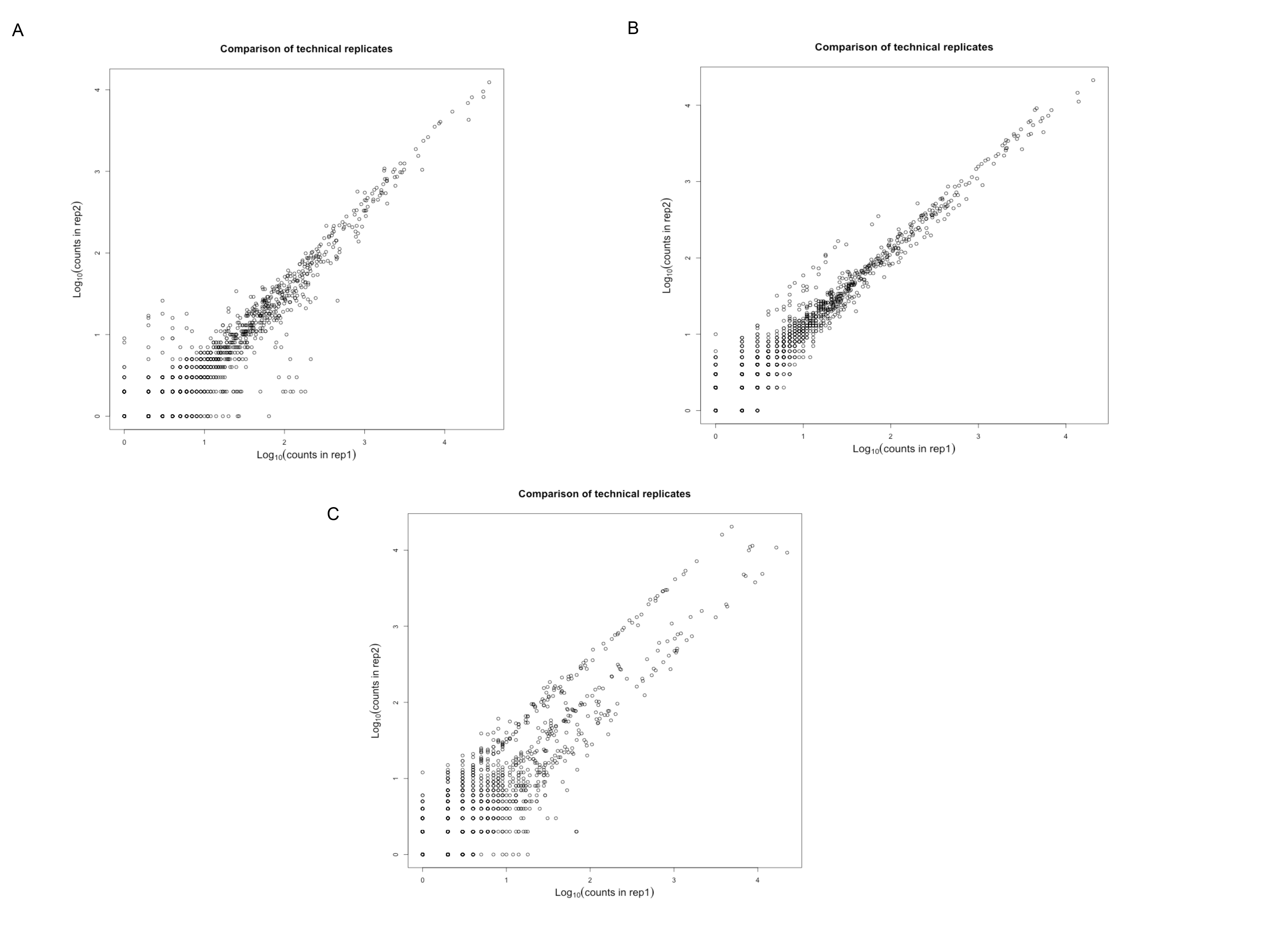

Supplement: S1 Fig — Variability was minimized by grouping samples by the plate on which they were sequenced. The Y axis displays log10 transformed raw read counts per OTU for the first replicate and the X axis displays these values for the second replicate. (A) Log10 transformed raw counts per OTU from Plate 2, samples sequenced January 2015. (B) Log10 transformed raw counts per OTU from Plate 1, samples sequenced January 2015. (C) Log10 transformed raw counts per OTU from Plate 3, samples sequenced December 2014. (TIF) [file pone.0173848.s001.tif]

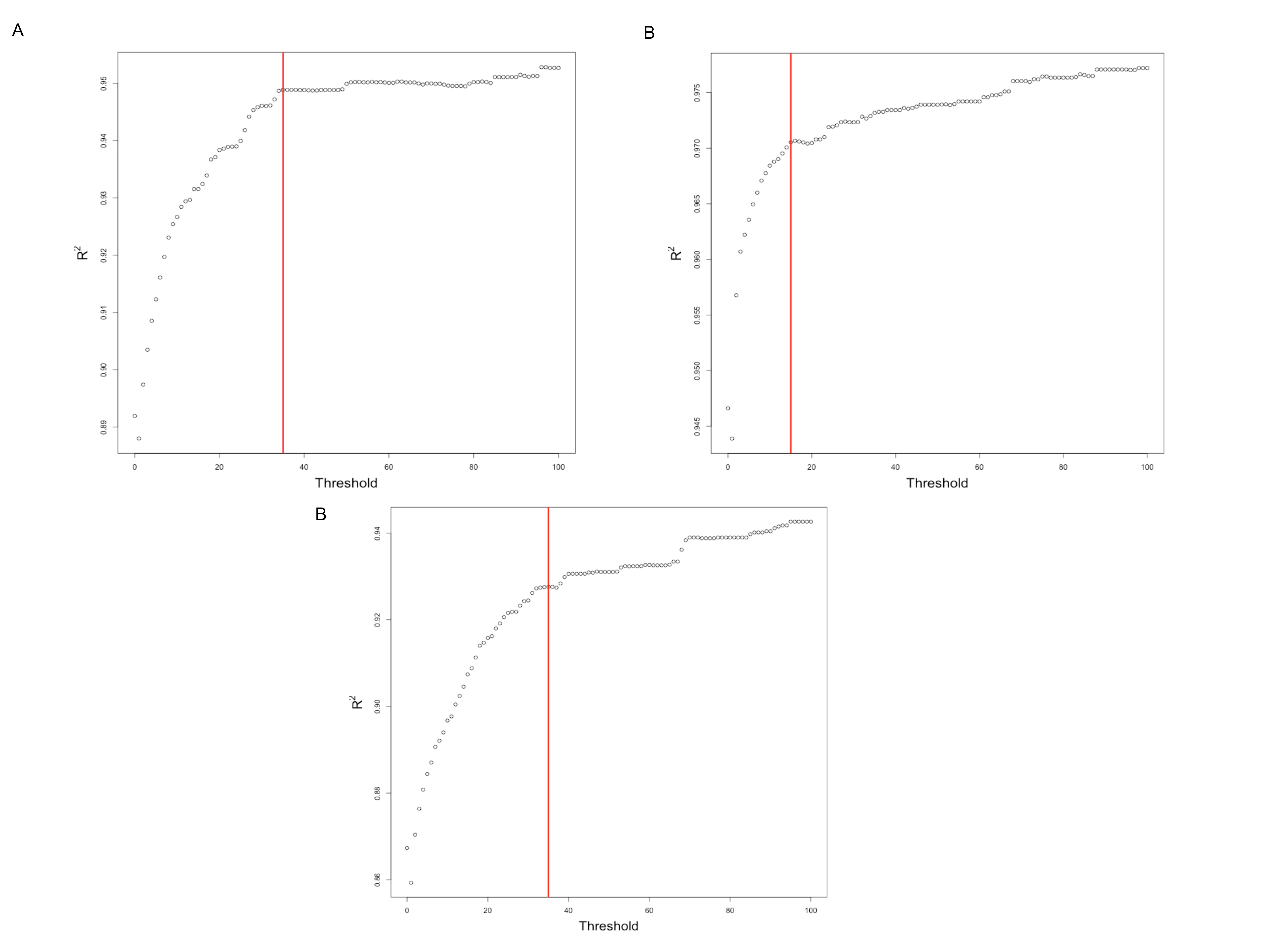

Supplement: S2 Fig — The threshold for log10 transformed raw counts per OTU per sequencing batch was set where the regression began to plateau (red line), indicating acceptable levels of read count correlation between the replicate samples. (A) R2 values for raw counts for samples on Plate 2, samples sequenced January 2015. (B) R2 values for raw counts for samples on Plate 1, samples sequenced January 2015. (C) R2 values for raw counts for samples on Plate 3, samples sequenced December 2014. (TIF) [file pone.0173848.s002.tif]

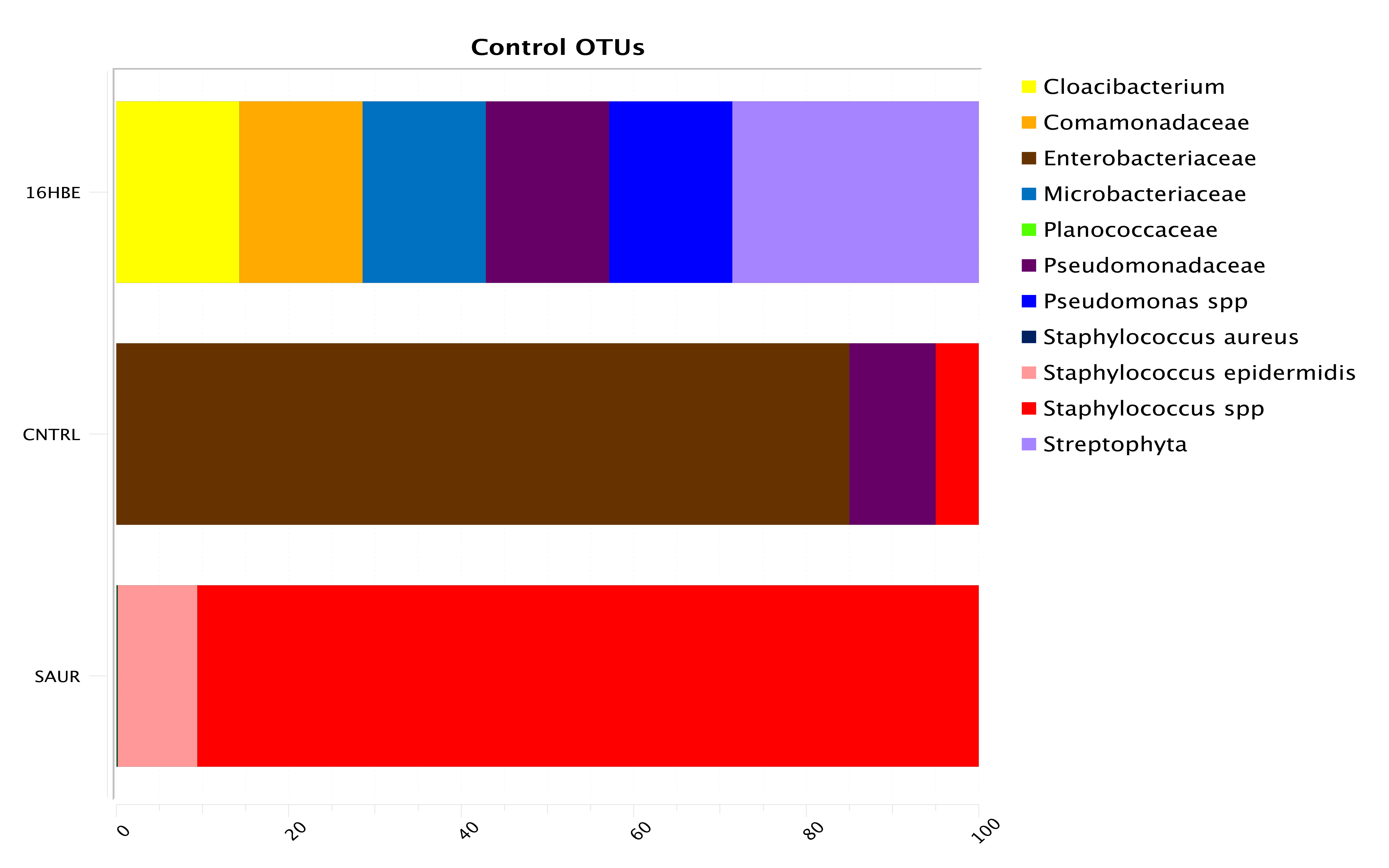

Supplement: S3 Fig — Each bar represents OTUs detected among human (16HBE), Staphylococcus aureus (SAUR), and reagent (CNTRL) DNA controls normalized to 100%. n = 6 (TIF) [file pone.0173848.s003.tif]

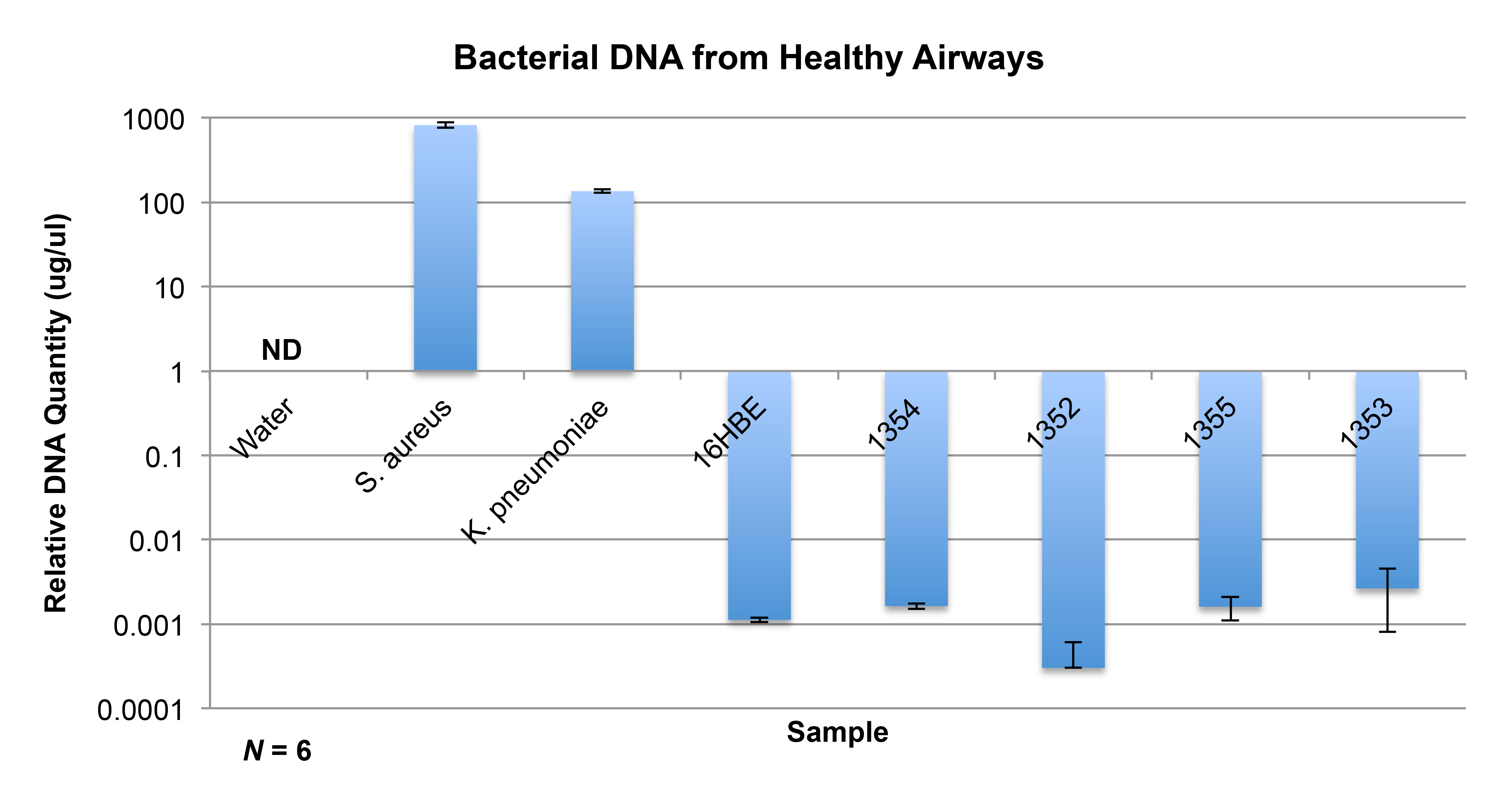

Supplement: S4 Fig — Bronchoscopy was performed on healthy volunteers and DNA was extracted from airway washings in the same manner as the burn patient samples. Extracted DNA was quantified using the universal primer set developed by Maeda et. al. DNA extracted from Staphylococcus aureus and Klebsiella pneumoniae were used as positive controls and DNA from the human cell line 16HBE was used as a negative control. A water-only reagent control was included as well. DNA extracted from six healthy volunteers did not contain significantly more DNA than the negative control. (n = 6). (TIF) [file pone.0173848.s004.tif]

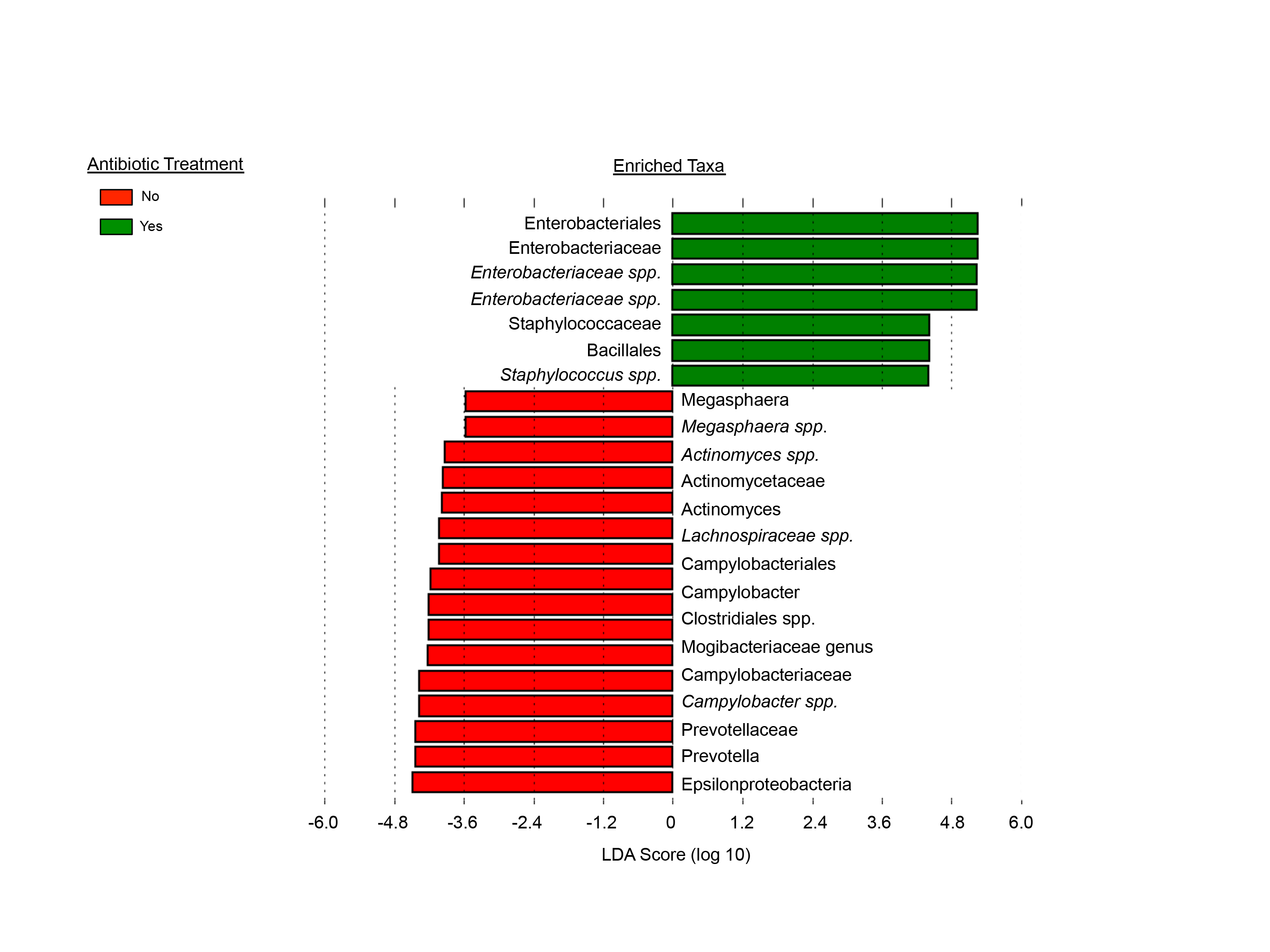

Supplement: S5 Fig — Specific taxa are enriched among non-hypoxemic patients who did and did not receive antibiotics. Analysis with LEfSe detects significant enrichment of bacteria in the Enterobacteriales order with antibiotic treatment, while several other taxa were enriched without treatment. (TIF) [file pone.0173848.s005.tif]
