# Supplementary material for: Alterations in airway microbiota in patients with PaO2/FiO2 ratio ≤ 300 after burn and inhalation injury
Source: PLoS One. 2017 Mar 30;12(3):e0173848. doi: 10.1371/journal.pone.0173848 (PMC5373524; doi:10.1371/journal.pone.0173848)
Supplement: S1 Table — (DOCX) [file pone.0173848.s006.docx]

**S1 Table. Distribution of Patient Samples by PaO_2_ /FiO_2_ Ratio Among Sequencing Plates.**

| PaO_2_ /FiO_2_ Ratio | Plate 1 | Plate 2 | Plate 3 |
| --- | --- | --- | --- |
| <300 | 6 | 11 | 7 |
| >300 | 6 | 14 | 4 |
