# Supplementary material for: Alterations in airway microbiota in patients with PaO2/FiO2 ratio ≤ 300 after burn and inhalation injury
Source: PLoS One. 2017 Mar 30;12(3):e0173848. doi: 10.1371/journal.pone.0173848 (PMC5373524; doi:10.1371/journal.pone.0173848)
Supplement: S2 Table — (DOCX) [file pone.0173848.s007.docx]

**S2 Table. Averaged Percent of Sequences for Positive and Negative**

**Controls.**

| **Control** | **Percent of Total Sequences** | **Percent of Total Molecule Tags** |
| --- | --- | --- |
| Human (16HBE) | 1.1 | 0.002 |
| *Staphylococcus aureus* (SAUR) | 1.2 | 2.3 |
| Reagent (CNTRL) | 0.44 | 0.002 |

Percent of averaged total sequences and molecule tags for human (16HBE; *n* = 2 ), *Staphylococcus aureus* (SAUR; *n* = 2) and reagent (CNTRL; *n* = 2) controls.
