# Supplementary material for: Alterations in airway microbiota in patients with PaO2/FiO2 ratio ≤ 300 after burn and inhalation injury
Source: PLoS One. 2017 Mar 30;12(3):e0173848. doi: 10.1371/journal.pone.0173848 (PMC5373524; doi:10.1371/journal.pone.0173848)
Supplement: S3 Table — (DOCX) [file pone.0173848.s008.docx]

**S3 Table. Patient Clinical Cultures.**

| Patient Number | Bacteria Cultured | Percent of Individual Community* | Antibiotic Treatment |
| --- | --- | --- | --- |
| 14 | ORSA | 0 | None |
| 79 | Unknown | NA | Vancomycin/iso-dex (1g/200ml IV) & piperacillin/tazobactam/isoOsmoticPMB (3.375g/ 50ml IV) |
| 93 | OSSA | 0.1 | None |
|  | *S. pneumoniae* | 54 |  |
| 104 | *S. pneumoniae* | 17.1 | Tigecycline 50mg IV q12h |
| 124 | *S. pneumoniae* | 35.3 | Tigecycline 50mg IV q12hr & piperacillin 3.375 q6hr |
| 128 | *Acinetobacter* | 0.02 | None |
|  | *H. influenzae* | 0 |  |
| 169 | *S. pneumoniae* | 0.1 | Piperacillin/tazobactam/isoOsmoticPMB (3.375g/ 100ml IV) |
| 202 | *Enterobacter* | 0.1 | Tigecycline 50mg IV q12h & piperacillin 3.375 q6hr |
|  | *H. influenzae* | 0 |  |
|  | *S. pneumoniae* | 0.1 |  |
|  | OSSA | 91.2 |  |
| 308 | Unknown | NA | Vancomycin hcl 450mg q8hr |
| 346 | OSSA | 0 | None |
|  | *S. pneumoniae* | 25 |  |
| 380 | Unknown | NA | Tigecycline 50mg IV q12h & piperacillin/tazobactam/isoOsmoticPMB (3.375g/ 100ml IV) |

Bacteria detected by clinical culture from bronchoscopy per patient, their corresponding abundance as detected by NGS, and patient antibiotic treatment.
