# Supplementary material for: Alterations in airway microbiota in patients with PaO2/FiO2 ratio ≤ 300 after burn and inhalation injury
Source: PLoS One. 2017 Mar 30;12(3):e0173848. doi: 10.1371/journal.pone.0173848 (PMC5373524; doi:10.1371/journal.pone.0173848)
Supplement: S1 File — (DOCX) [file pone.0173848.s009.docx]

Online Data Supplement

Methods

**DNA Extraction and Quantification**

Samples were centrifuged to separate the supernatant from the cellular fraction and these were stored separately at -80°C. The cellular fraction was used to extract bacterial DNA, and these were thawed briefly in a water bath at 35°C prior to extraction. Both enzymatic and physical methods were used to lyse the bacterial cell walls; the samples were resuspended in a lysis buffer including lysozyme and were placed in a Vortex mixer for 10 minutes. Samples were treated with RNase A to degrade contaminating RNA and DNA was extracted using the Qiagen QIAmp UCP Pathogen Mini Kit according to the manufacturer’s protocol. The recommended lysis step was skipped in favor of the method described above. Quantitative real-time polymerase chain reaction (qPCR) was used to quantify human DNA[1] in the samples and total DNA was quantified using the Applied Biosystems PicoGreen double-stranded DNA dye. Bacterial DNA was quantified indirectly by subtracting the quantity of human DNA from total DNA.

**Quantification of Bacterial DNA from Airways**

Strains of *S. aureus* and *K. pneumoniae* used as standard curve DNA were received from Carolina Biologicals (Burlington, NC). The 16HBE14o- cell line was a gift from D.C. Gruenert.[2] DNA extraction for all samples and standards was done as described above. Bacterial DNA was quantified using primers designed by Maeda *et. al.*[3].

**Sequencing Data and Statistical Analysis**

Quality trimming of the resulting sequencing reads was performed using the Illumina CASAVA software. The MTToolbox pipeline, developed specifically to handle sequences resulting from the molecule tagging method, was used to generate consensus sequences from the molecule tags, group them into operational taxonomic units (OTUs), and match them to the GreenGenes 16S rRNA gene database to identify the sequences to the lowest bacterial taxonomic level possible[4,5].

References

1. McCullough SD, Xu X, Dent SYR, Bekiranov S, Roeder RG, Grant P a. Reelin is a target of polyglutamine expanded ataxin-7 in human spinocerebellar ataxia type 7 (SCA7) astrocytes. Proc Natl Acad Sci U S A. 2012;109: 21319–24. doi:10.1073/pnas.1218331110

2. Cozens AL, Yezzi MJ, Kunzelmann K, Ohrui T, Chin L, Eng K, et al. CFTR expression and chloride secretion in polarized immortal human bronchial epithelial cells. Am J Respir Cell Mol Biol. 1994;10: 38–47. doi:10.1165/ajrcmb.10.1.7507342

3. Maeda H, Fujimoto C, Haruki Y, Maeda T, Kokeguchi S, Petelin M, et al. Quantitative real-time PCR using TaqMan and SYBR Green for Actinobacillus actinomycetemcomitans , Porphyromonas gingivalis , Prevotella intermedia , tetQ gene and total bacteria. FEMS Immunol Med Microbiol. 2003;39: 81–86. doi:10.1016/S0928-8244(03)00224-4

4. Yourstone SM, Lundberg DS, Dangl JL, Jones CD. MT-Toolbox: improved amplicon sequencing using molecule tags. BMC Bioinformatics. 2014;15: 284. doi:10.1186/1471-2105-15-284

5. DeSantis TZ, Hugenholtz P, Larsen N, Rojas M, Brodie EL, Keller K, et al. Greengenes, a Chimera-Checked 16S rRNA Gene Database and Workbench Compatible with ARB. Appl Environ Microbiol. 2006;72: 5069–5072. doi:10.1128/AEM.03006-05
